# Development of Quantitative Rapid Isothermal Amplification Assay for *Leishmania donovani*

**DOI:** 10.3390/diagnostics11111963

**Published:** 2021-10-22

**Authors:** Md Anik Ashfaq Khan, Khaledul Faisal, Rajashree Chowdhury, Prakash Ghosh, Faria Hossain, Manfred Weidmann, Dinesh Mondal, Ahmed Abd El Wahed

**Affiliations:** 1 Institute of Animal Hygiene and Veterinary Public Health, University of Leipzig, An den Tierkliniken 1, 04103 Leipzig, Germany; anik.ashfaq@gmail.com; 2 Nutrition and Clinical Services Division, International Centre for Diarrheal Disease Research, Dhaka 1212, Bangladesh; otivuj@gmail.com (K.F.); chowdhury_rajashree@yahoo.com (R.C.); dreamix888@gmail.com (P.G.); faria109@gmail.com (F.H.); 3 Institute of Microbiology & Virology, Brandenburg Medical School, 01968 Senftenberg, Germany; manfred.weidmann@mhb-fontane.de; 4 Laboratory Sciences and Services Division, International Centre for Diarrheal Disease Research, Dhaka 1212, Bangladesh

**Keywords:** isothermal amplification, quantitative molecular assay, recombinase polymerase amplification, point-of-need testing, leishmaniasis

## Abstract

Quantification of pathogen load, although challenging, is of paramount importance for accurate diagnosis and clinical management of a range of infectious diseases in a point-of-need testing (PONT) scenario such as in resource-limited settings. We formulated a quantification approach to test the standard-curve based absolute quantification ability of isothermal recombinase polymerase amplification (RPA) assay. As a test of principle, a 10-fold dilution series of *Leishmania donovani* (LD) genomic DNA prepared in nuclease-free-water (NFW), and from culture-spiked-blood (CSB) were tested, and a 15 min assay was performed. A modified algorithm was formulated to derive the detection outcome. The threshold-record times (Tr) in seconds thus obtained were plotted against the initial load of parasite genomes for log-linear regression analysis. The quantitative RPA (Q-RPA) assay was further evaluated against a LD quantitative (q)-PCR assay with DNA extracted from visceral and post-Kala-azar dermal leishmaniasis case specimens and stratified into different ranges of threshold cycle (Ct). The best-fitted regression models were found linear with mean r^2^/root mean square error (RMSE) values of residual points (in seconds) estimated as 0.996/8.063 and 0.992/7.46 for replicated series of NFW and CSB, respectively. In both series, the lower limit of detection reached less than 0.1 parasite genome equivalent DNA. Absolute agreement between Q-RPA and LD-qPCR was found for test positivity, and strong positive correlations were observed between the Tr and Ct values (r = 0.89; *p* < 0.0001) as well as between the absolute parasite loads (r = 0.87; *p* < 0.0001) quantified by respective assays. The findings in this very first Q-RPA assay for leishmaniasis are suggestive of its potential in monitoring LD load in clinical specimens, and the development of rapid Q-RPA assays for other infectious diseases.

## 1. Introduction

Modern nucleic acid testing (or NAT) approaches such as polymerase chain reaction (PCR) have led to a considerable increase in diagnostic sensitivity, which often outperforms culture-based approaches [1]. However, the fundamental yet unresolved issue of whether mere detection of pathogens can be interpreted for the clinical outcome, either in a well-established diagnostic microbiological laboratory or point-of-need testing (PONT) scenario, remains a challenge. Furthermore, monitoring of pathogen load post-infection can be relevant for clinical management [2], deployment of treatment initiatives [3], drug efficacy and pathogen resistance [4,5], pathogen proliferation kinetics for early detection of disease [6], and therefore relevant for clinical outcome [7,8,9]. Thus, while qualitative diagnostics are the mainstay of diagnosis of most infectious diseases, the development and implementation of quantitative molecular diagnostic assays is of prime importance for a growing number of treatable infectious diseases.

Real-time quantitative (q)-PCR, which employs a time-to-signal based relationship of a specific threshold concentration of amplified products with the initial copy number of the template (i.e., number of cycles or Ct), is the most widely studied approach in the quantification of the pathogen load in biological specimen. Estimation of the amount of initial template concentration by real-time qPCR can be performed either by relative or absolute quantification [10]. For quantitative nucleic acid analysis methods to be used at the point of need, such as in resource-limited settings, among desired characteristics are simplicity in operation, low-cost and feasibility. However, due to specific requirements like cyclic heating and cooling systems and thermally stringent materials, PCR reactions within an enclosed microfluidic system- be it in a conventional or real-time apparatus- render an imperfect solution for incorporation into PONT platforms. Although, efforts have gone into necessary optimization and miniaturization of the assay as well as detection systems onboard [11,12,13], it would be advantageous to focus on detection methods that are less expensive and inherently simple, miniaturizable, robust, and portable for PONT molecular diagnosis. Thus, research has turned towards isothermal nucleic acid amplification techniques [14] that mainly differ from a typical PCR reaction in the strand separation mechanism. Importantly, determination of pathogen load is a desirable characteristic of isothermal technologies for PONT integration [15] and this in response, led to progresses in quantitative strategies for isothermal assay formats such as nucleic acid sequence based amplification (NASBA), loop-mediated amplification (LAMP) and recombinase polymerase amplification (RPA) [16,17,18,19,20].

*Leishmania* is an intracellular parasite that causes a range of pathologies involving cutaneous and visceral organs of human and animals. Among several clinically important species, *L. donovani* (LD) typically causes visceral leishmaniasis (VL/Kala-azar) which is often fatal if left untreated, and post-Kala-azar dermal leishmaniasis (PKDL)- a skin complication that may lead to deformity. Although these neglected tropical disease forms are mainly endemic in the Indian subcontinent, however increasing evidences suggest the presence of co-endemic and imported *Leishmania* species [21,22,23]. Over the last decade a variety of molecular tools including conventional and real-time qPCR, and isothermal based methods such as LAMP, RPA, and NASBA assays have been developed in the search of appropriate tools for the detection of different species of *Leishmania* [20,24,25], but quantification has been confined to real-time qPCR. The quantification of pathogen load in leishmaniasis has been suggested to be helpful to monitor drug response, parasite kinetics and infection dynamics. For instance, a prospective study to determine the efficacy of two formulations of amphotericin B suggested significant correlation between parasite load and clinical outcome in VL patients, while monitoring of parasite kinetics signified definitive cure with a parasite load as low as below 2 parasites/mL of blood [26]. On the other hand, a threshold load of 1 parasite/mL of blood was observed to distinguish asymptomatic carriage from disease development [27], whereas, a cut-off of 10 parasites/mL blood or lower can indicate an increased chance of disease relapse post-treatment [26,27,28,29]. In an earlier study, we showed that a real-time qPCR assay for LD (LD-qPCR) could successfully differentiate cured VL and PKDL from active and/or relapsed disease forms- rendering the potential of quantification of parasite load as a prognostic marker of VL and PKDL [30]. Nevertheless, RPA offers several advantages over other molecular tools in terms of feasibility, time to result, simplicity in primer design, capability to target longer sequences, tolerance to inhibitors, cost and dispensability of a heating source. It facilitated development of detection assays for a number of pathogens of human and animal importance [31]. For the detection of LD in clinical specimens, with the broad scope of its deployment in the endemic settings where diagnostic establishment of PCR based applications can be challenging, we have previously developed a qualitative LD-RPA assay [24], which has recently completed its phase II diagnostic trial [32]. Monitoring of LD load in patients during diagnosis and in treatment follow-ups can be clinically significant (e.g., drug dosage and efficacy testing, relapse and cure assessment etc.) and may facilitate assessing the prevalence of the potentially transmitting population in resource-poor endemic settings, however implementation of qPCR for this purpose remains farfetched.

Quantitative LD-RPA as a plausible alternative for PONT diagnosis and prognosis in field conditions could be a very powerful tool. Here we describe an absolute RPA quantification assay for LD. Overall, our observations are in support of the potential of the quantitative RPA assay to detect and monitor LD infection in leishmaniasis patients.

## 2. Materials and Methods

### 2.1. Theoretical Considerations

The RPA process initiates in a phase separated system formulated by a viscous crowding agent which prompts the reactions to occur within colloidal globules that localize RPA enzymes, nucleotides and template molecules [33]. We assume that this compartmentalized reaction principle of RPA can be harnessed to explain template-time relationship from unperturbed amplification data unlike the default (classical) RPA method that incorporates a mixing step [34]. We further considered that the template-time relation is more likely to be synchronized regardless of template load at the early growth phase of RPA reaction where the limited supply of DNA determines the initial rate of amplification and initiates a roughly exponential trend. Due possibly to the diffusion mediated effects [35,36] on reaction space and time, the template-time relationship becomes progressively nonsynchronous in the continuity of growth phase (Figure S1). Several methodological variations have been formulated to date in order to increase RPA amplification efficiency. The comparative features of these approaches as well as the theoretical considerations behind our methodological approach are highlighted in the Supplementary Materials (Supplementary S1 and Figure S2).

### 2.2. Sample Source

The samples that were tested in this study comprise of: (1) LD-qPCR positive archived DNA extracted from specimens collected from VL (blood; *n* = 30) and PKDL (skin biopsy; *n* = 17) cases; (2) LD-qPCR negative archived DNA samples extracted from specimens collected from asymptomatic (blood; *n* = 30) subjects, who were positive in rk39 rapid immunochromatographic test (ICT; InBios International Inc., Seattle, WA, USA) or direct agglutination test (DAT) for LD bodies; (3) standard DNA of a WHO reference strain of LD (*L. donovani* MHOM/IN/80/DD8); and (4) a blood sample spiked with VL patient-derived and in-house cultured promastigotes in a serial dilution series. Archived samples were obtained from the Ethical Review Committee, icddr,b, approved study (PR-18023), in which VL and PKDL patients were enrolled and provided informed consent regarding the use of the anonymous samples in further research.

### 2.3. DNA Standard Preparation

Serial dilutions of standard DNA of *L. donovani* MHOM/IN/80/DD8 strain were prepared in nuclease free water (NFW) from 1 ng to 1 fg of parasite DNA corresponding to 10^4^ to 10^−2^ parasites per reaction of RPA (100 fg equals 1 parasite). For preparation of promastigote culture spiked in blood (CSB), approximately 10^6^ LD parasites were taken and centrifuged at 6000 rpm for 5 min. One ml of PBS (0.02 M, pH 7.2) was added to wash the pellet followed by centrifugation (6000 rpm for 5 min). The pellet was dissolved in 200 µL of PBS (i.e., 10^6^ parasites/200 µL). Twenty µL was added to 180 µL of LD-free human blood to make a final volume containing 10^5^ parasites, which was serially diluted (1:10) in blood to obtain 10^4^ to 10^0^ parasites in 200 µL of blood. This corresponded to 2.5 × 10^2^ to 2.5 × 10^−2^ parasite equivalent genomic DNA per reaction of RPA (described below). DNA from spiked samples were extracted with DNeasy Blood and Tissue kit (Qiagen, Hilden, Germany).

### 2.4. RPA and Real-Time qPCR Assays

The classical RPA (hereby referred to as or C-RPA) was performed using the TwistAmp exo kit (TwistDx Ltd., Cambridge, UK) in a 50 μL volume (5 μL template DNA and 45 μL mastermix) as per manufacturer’s recommendation, and according to our previous protocol for LD-RPA development [24]. In brief, after preparing the mastermix, all components were added in the lid of the reaction tube leaving the pellet undissolved. The tubes were then vortexed and centrifuged at once and placed into the Axxin T8 fluorescence reader (Axxin, Fairfield, VIC, Australia) at 42 °C/15 min with 10 or 20 s read-time intervals. After 4 min, the tubes were removed for mixing and brief centrifugation, and then placed back into the reader. Each run included one reaction with molecular grade water as a negative control. Fluorescence was detected in the FAM channel (470 nm excitation and 520 nm emission). In addition to the classical protocol, a modified protocol was performed (hereby termed as quantitative RPA or Q-RPA). In brief, the temperature was set to 40 °C for an uninterrupted assay run. Dissolving of the lyophilized enzyme and the nucleotide mix in each tube was ensured before placing it into the Axxin device. The primer and probe sequences used in either assay targeted the kinetoplast minicircle DNA (GenBank accession no. Y11401.1) which has around 10,000 copies in a single LD cell.

LD-qPCR was performed as described [30]. To quantify the parasite load in each sample, every run included a standard curve generated by assaying serially diluted standard LD DNA that correspond to 10^4^–10^−1^ parasites per reaction.

### 2.5. Curation of Q-RPA Amplification Data for Detection and Quantification

The observable template amplification phase denotes positive change in the acquisition of total fluorescence number over time for a consecutive period of read-points, denoted hereby as Ap. We assume that the Ap separates the background/noise floor. Noise-unbiased sample fluorescence (F_n_) at any read-point was thus derived and it was used to calculate the fold change ratio in fluorescence acquisition rate (R_fold_) for any given read-point above the noise floor. For the reaction to assert exponential behavior, R_fold_ must be valued >1.0 for a period of consecutive read-points, denoted hereafter as E-phase. The threshold record-time (Tr) would thus correspond to a time point that belongs to E-phase. The optimal cut-off duration (in seconds) of E-phase was then determined by using LD-qPCR positive (*n* = 30; Ct range: 30.8 to 38.9) and LD-qPCR negative (*n* = 30) DNA to differentiate between a true template amplification from template negative outcome. To generate standard curve from serial dilution series of parasite genome, Tr value of each dilution was estimated from an arbitrary F_n_ value (F_n(arbitrary)_, which is an integer) that is reached within the E-phase by all the respective dilutions. Standard curves were generated from the log-linear regression analysis after plotting Tr values against the parasite loads that represent corresponding dilution. A total of 37 DNA samples extracted from VL (*n* = 20; blood) and PKDL (*n* = 17; skin biopsy) clinical specimens were blindly evaluated by C-RPA, Q-RPA and LD-qPCR for comparison. In the Q-RPA and LD-qPCR assay, threshold times and absolute template number were extrapolated from the standard curve.

Furthermore, for generalization of the exponential amplification that is assumed to initiate about the Tr under a hypothetically constant reaction condition, the normalized fluorescence reads starting about the F_n(arbitrary)_ value were transformed as a function of Tr to fit to a deviated model of what has been previously described mathematically [35]. The details of the detection and quantification algorithm are given in the Supplementary Materials (Supplementary S2).

### 2.6. Statistical Analysis

The sensitivity and specificity of the Q-RPA assay against C-RPA and LD-qPCR assays were calculated by standard formula [37]. Depending on the distribution of data, pairwise comparisons were performed between threshold points of C-RPA (Tt) and Q-RPA (Tr), and between the absolute parasite loads estimated by Q-RPA and LD-qPCR. Further, correlation analysis was performed among Tr (Q-RPA), Ct (LD-qPCR) and Tt (C-RPA) and respective correlation coefficients (r-value) were determined. Variability in the assay dilution series was reported as the coefficient of variation (CV; shown as the ratio of mean to standard deviation (SD) in Tr values for each of the dilution points of the replicated dilution series. A *p*-value < 0.05 was considered as an indication of statistically significant result. Statistical analysis and regeneration of plots and curves were performed using SPSS v20 and Graphpad Prism v8.0.

## 3. Results

### 3.1. Generalization, Linearity and Reproducibility of Q-RPA Amplification

The reader generated fluorescence values were used to estimate the acquisition period (Ap), based on which normalization was performed to synchronize noise level. This was followed by estimation of an exponential amplification phase (E-phase) and threshold record-time (Tr) that lies within the early E-phase (i.e., Tr ∈ E-phase ∈ Ap). In determining linearity of the template-time relationship, several unique F_n(arbitrary)_ values within the E-phase of individual dilutions were tested to estimate Tr, which were then plotted against the initial parasite genome number. The log-linear model was chosen based on the goodness-of-fit (r^2^) of the Tr curve with the least root mean square error (RMSE) values of the residual Tr points.

**Figure 1:**
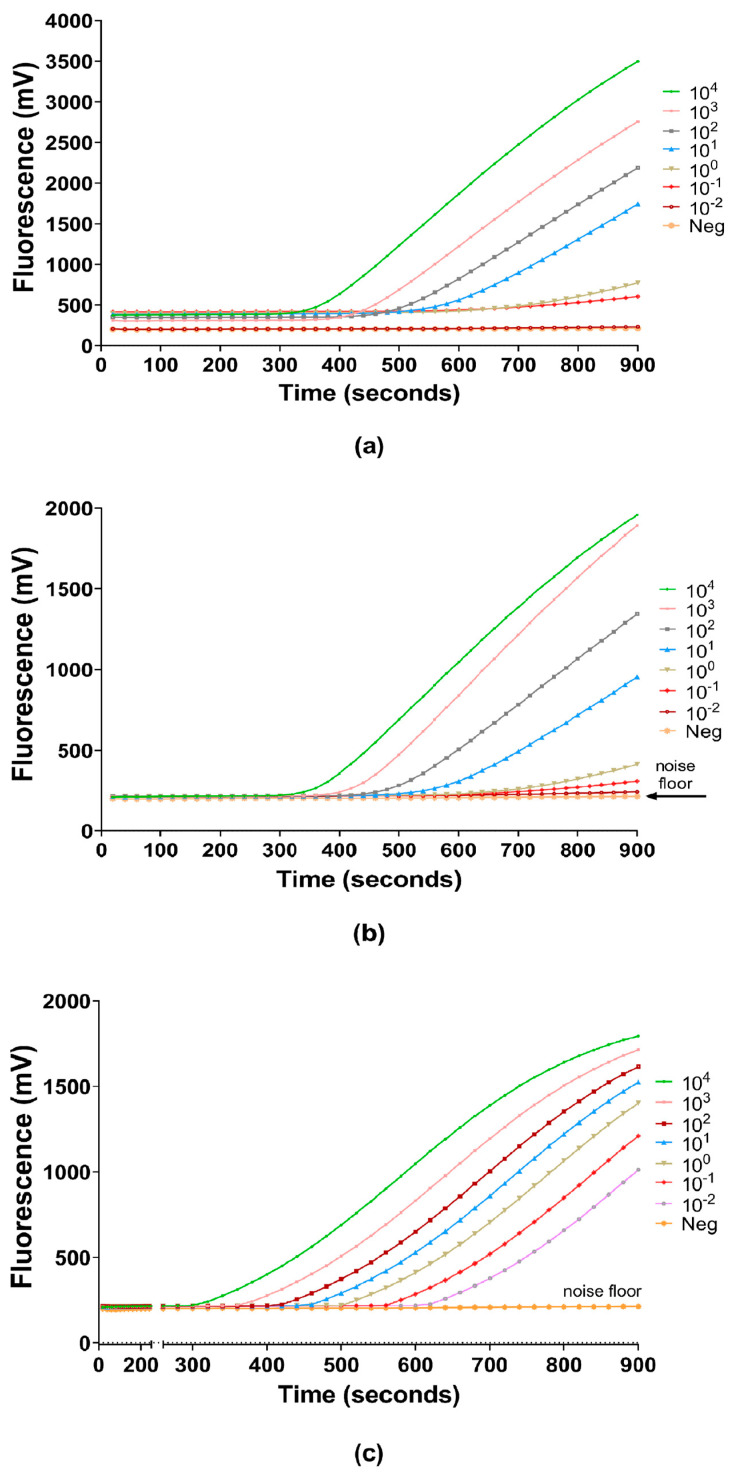
RPA amplification curves of NFW dilution series (10^4^ to 10^−2^ genome/reaction) in terms of relative fluorescence acquisition in a 15 min (900 s) run protocol. Plots were generated by using (**a**) machine read data; (**b**) normalized data; (**c**) transformed data to generalize the amplification.

A simplified model of the RPA amplification process was thus generated by transformation of normalized data under the assumption that Tr is the starting point of exponential growth and, under ideal assay conditions, each of the reactions regardless of dilution is consistent in amplicon generation rate until reaching the fluorescence maxima. In this case, the relative fluorescence accumulated at the terminal end of the reaction by the lowest dilution was the maximal fluorescence number. The RPA amplicon generation rate (k) was derived by taking the average of R_fold_ values that belong to the E-phase of all the dilution series samples, and thus a simplistic plot of the RPA amplification was obtained (Figure 1).

**Figure 2:**
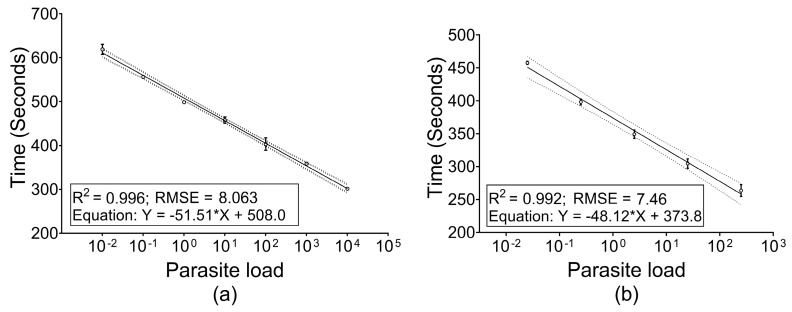
Performance of Q-RPA for obtaining standard curve. Tr values are plotted from replicated assay runs against parasite load equivalent genomic DNA. The plot represents Tr values and parasite load fitting a linear function. (**a**) DNA extracted from reference strain and diluted in NFW in the range of 10^4^ to 10^−2^ parasite per reaction and assayed in duplicate (**b**) DNA extracted from parasite spiked blood to give a range of 2.5 × 10^2^ to 2.5 × 10^−2^ parasite per reaction and assayed in triplicate.

The standard curve generated by a 10-fold serial dilution series of reference strain DNA in NFW had a linear dynamic range over 7 log-steps (10^4^ to 10^−2^ parasite genomes) with mean r^2^/RMSE values estimated as 0.996/8.063. On the other hand, for the CSB dilution series, linearity was found for 5 log-steps (2.5 × 10^2^ to 2.5 × 10^−2^ parasite genome) with mean r^2^/RMSE values of 0.992/7.46 (Figure 2). There was no significant difference (*p*-value = 0.16) in the slope between NFW and CSB dilution series and neither among the replicates of individual series. However, significant difference (*p*-value < 0.0001) was observed in the elevation between the NFW and CSB series. Reproducibility of the linearity was assessed as inter-assay variation of Tr values for the same dilution series in independent runs. Inter-assay mean coefficients of variations (CV) were found to be 1.19% (95% CI: 0.07–2.32%) and 1.84% (95% CI: 0.46–3.21%) for NFW and CSB series, respectively, indicating high reproducibility (Table 1).

### 3.2. Threshold E-Phase and Limit of Detection (LOD)

**Figure 3:**
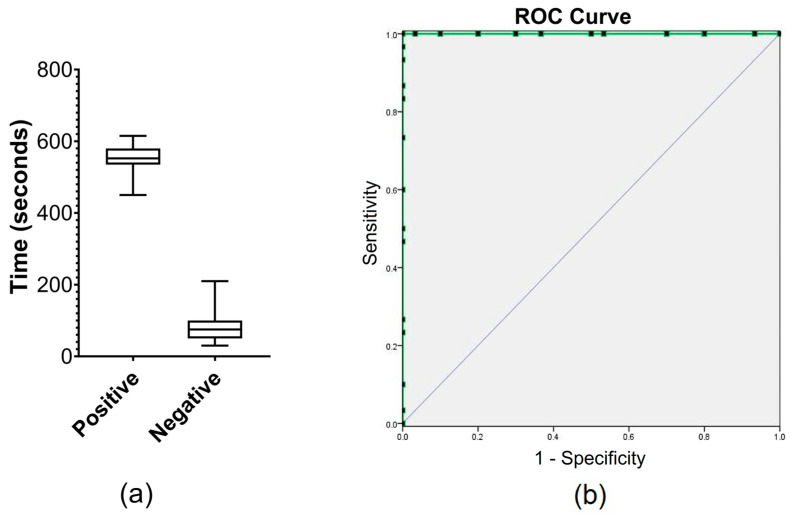
E-phase threshold determination. (**a**) Comparison between LD-qPCR positive and LD-qPCR negative samples in E-phase duration (in seconds) of Q-RPA. (**b**) Receiver-operating-characteristic curve (AUC = 1.00, *p* < 0.0001) for E-phase as the predictor of positive results by Q-RPA. Coordinates of the curve (green) are indicated by black dots.

The LD-qPCR positive case samples (*n* = 30) distributed around high to low Ct values and the LD-qPCR negative asymptomatic samples (*n* = 30) were used to test whether the E-phase (i.e., the longest consecutive period where R_fold_ > 1.0) can be a classifier for positive RPA detection in the sample pool (Table S1). It was found that LD-qPCR positive samples had a significantly higher duration of the E-phase at any period of reaction than LD-qPCR negative samples (median values: 552.5 vs. 75, *p* < 0.0001). The estimated threshold of 330 s predicted both sensitivity and specificity to reach 100% (95% CI: 88.65–100%) (Figure 3). If this threshold is applied to the standard dilution series, the lower LOD for the NFW dilution series would be 0.1 parasite equivalent genomic DNA, while it would remain 0.025 parasite equivalent genomic DNA for CSB series (Table 1).

### 3.3. Q-RPA Quantification and Comparison with C-RPA and LD-qPCR

Reproducibility of the Q-RPA assay and its quantitative ability were assessed blindly. For this, 17 out of 30 LD-qPCR positive samples that were assayed in the E-phase cut-off determination step were taken randomly along with 20 additional LD-qPCR positive samples ranging from high to low Ct values (Table S2). A 10-fold dilution series of standard genomic DNA (*L. donovani* MHOM/IN/80/DD8) was contemporarily assayed. The dilution series thus prepared for both Q-RPA and LD-qPCR assays with adjustment of reaction volumes resulted in a detection limit of 0.1 parasite equivalent genomic DNA per reaction in either assay (not shown). The resultant sample Tr each corresponded to the unique F_n(arbitrary)_ value set to generate the standard linear curve of the dilution series.

**Figure 4:**
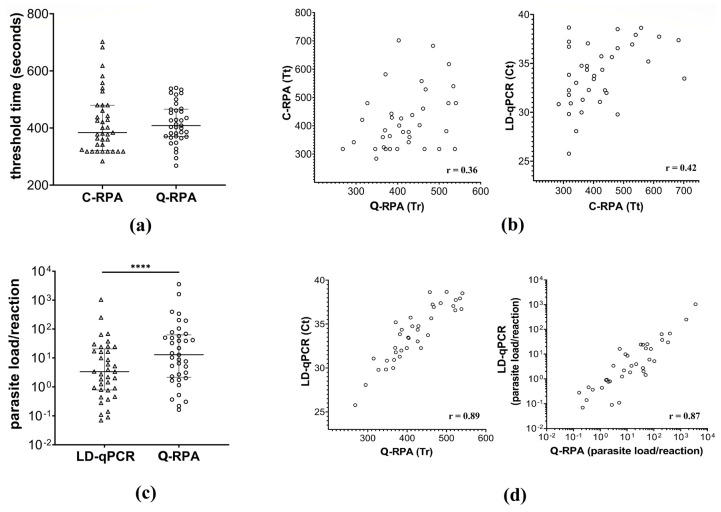
Quantitative performance of Q-RPA assay. (**a**) Threshold time difference between C-RPA (Tt) and Q-RPA (Tr); (**b**) Correlation plots for Tr vs. Tt and Tt vs. Ct; (**c**) Absolute parasite load per reaction extrapolated from LD-qPCR and Q-RPA standard dilution curves, **** *p* < 0.0001; (**d**) Correlation plot for Tr vs. Ct and relative parasite loads between LD-qPCR and Q-RPA assays.

The Q-RPA assay successfully detected all the 37 test samples, rendering 100% (95% CI: 88.43–100.00%) sensitivity against LD-qPCR. When C-RPA was performed on the given sample pool, it was found as well to have perfect concordance (Cohen’s kappa: 1.00; 95% CI: 0.93 to 1.00) with Q-RPA for the detection of LD. No significant difference (Wilcoxon matched-pairs signed rank test, *p* = 0.70) between the threshold times of C-RPA (Tt) and Q-RPA (Tr) was found, however, poor association (Spearman correlation, r = 0.36; 95% CI: 0.02–0.62) was observed between them. On the other hand, a significant pairwise association (*p* < 0.0001) was observed between Tr and Ct with an r-value of 0.89 (Spearman correlation; 95% CI: 0.80–0.95). When absolute parasite load (as genome copy number) per reaction was extrapolated from the standard curves of respective Q-RPA and LD-qPCR assays, there was a significant difference (Wilcoxon matched-pairs signed rank test, *p* < 0.0001) between the parasite loads reported by the two assays, however, strong positive association (Spearman correlation, r = 0.87; *p* < 0.0001) was observed between respective parasite loads, which indicates that the absolute quantitative ability of Q-RPA assay based on standard curve is comparable to that of quantitative PCR (Figure 4).

## 4. Discussion

Monitoring of parasite load in clinical samples can be advantageous for both clinical management and epidemiological assessment of leishmaniasis, a neglected tropical disease. In this regard, however, establishment of a field-deployable quantitative molecular tool in the resource-limited endemic settings remains a challenge. In this study, a quantitative RPA assay was developed and its performance assessed with clinical samples. Quantification was assessed using genomic DNA of LD and archived DNA extracted from specimens derived from VL and PKDL patients. In order to perform the Q-RPA assay, several modifications were introduced in the assay procedure as well as in the analytical method to derive results. The Q-RPA assay was allowed to record fluorescence without interruption for the entire assay duration omitting one additional step of mixing and centrifugation. As another deviation from the C-RPA approach [24], the exponential trend in amplification was used to define the output (positive or negative) rather than arbitrarily assigning a threshold in the derivative plot. Furthermore, continuous fluorescence recording allowed to derive a standard Q-RPA curve from the noise-normalized data to extrapolate initial template load of test samples. Quantification features (threshold time and parasite load/reaction) were evaluated on archived clinical samples against our previously developed LD-qPCR assay [30]. Strong correlations in threshold time and parasite load between Q-RPA and LD-qPCR assays as observed in this study indicate the potential of the Q-RPA assay as an alternative approach to quantify LD in clinical specimens.

A likely challenge with regards to our approach is defining background/noise and exponential phase in the reaction- two key elements in the polymerase based quantification principle. In the recombinase filament-induced delay time period preceding the growth phase, the signal is actually small but non-zero. This noise floor is generated by random noise processes possibly arising from instrumentation-specific stochastic factors, inefficient fluorophore quenching, primer-dependent artefacts, and due to sensitivity of the signal to matrix effects [35,38]. Our earlier observation suggests that noisy fluorescence is prone to variability even between replicates of the same assays (results not shown). Therefore, before estimating the exponential reporting window, sample-to-sample (or reaction-to-reaction) variation in noise level was essentially minimized by normalization of individual noise to true background noise derived from the non-template control. Next, the Q-RPA reporting window should have a measurable trend of amplification (i.e., fluorescence level) for template-contained samples that is quantitatively different from that of non-template samples. In this study, this trend was defined for the given reporting interval period (i.e., 10/20 s) as the rate of amplicon generation (i.e., fluorescence acquisition) at a given read-point surpassing the rate obtained at its former read-point in a continuous manner for a cut-off reporting window (i.e., exponential phase or E-phase). Although one of the challenges of the mixing free reaction has been attributed to the delay in producing a result [39], it was shown in the present study that by defining the exponential trend within the context of a reporting window of the E-phase, template load of around 100 copies of LD kDNA in the NFW dilution series could be detected within 11 min.

Since the goal of this study was to evaluate RPA quantification in clinical samples of leishmaniasis, the cut-off reporting window of the exponential phase (E-phase) was determined empirically using LD-qPCR positive (high to low Ct values) and LD-qPCR negative clinical samples. The estimated E-phase cut-off suggests that exponential amplification of at least 330 s can differentiate between a positive and negative outcome with good confidence. However, low number of samples used in the estimation of the E-phase cut-off has possibly downgraded the detection limit of NFW series to 0.1 parasite equivalent genomic DNA per reaction in this study, as the standard curves were considerably linear up to 0.01 parasite equivalent genomic DNA. This limit is nevertheless in congruence with our previously developed and clinically evaluated qPCR assay for LD [30]. Furthermore, the LOD of the CSB dilution series was investigated to evaluate the effect of non-template background DNA and sample matrix on the linearity of the RPA amplification trend, particularly for low template reactions. In the CSB series it was observed that RPA amplification trends follow the template-time relationship in a similar manner as in the NFW series (no significant difference in standard curve slopes). However, the LOD of the CSB series was a bit higher (0.025 parasite equivalent genomic DNA per reaction) than that of the NFW series. Nonetheless, the CSB series was restrained by the use of promastigotes spiked in blood, and therefore further dilution up to a fraction of a promastigote was not possible. Interestingly, the CSB series with a similar dilution range tended to reach the Tr much faster resulting in significantly higher elevation of the standard curve than that of the NFW series. This hints at possible strain-specific differences in kDNA copy numbers (i.e., reference LD strain in NFW series vs. in-house clinical strain in CSB series). At the same time, the RPA template-time relationship does not seem to be perturbed in the presence of sample matrix/background DNA. Moreover, there were no significant differences among the respective slopes for the replicate runs of the NFW and CSB dilution series. 

A number of real-time qPCR assays that target different genetic markers of *Leishmania* species and sample types have been developed to date for use in veterinary or human medicine, as discussed elsewhere [40]. Owing to the conserved sequence of the 18S rDNA, and the heterogeneity as well as the high copy number of minicircle kDNA, these target sequences allow detection at the genus or subgenus level with resolution as low as fractions of parasite equivalent genomic DNA [41]. This is particularly beneficial for quantification of parasite load in cases where enumeration of parasitemia is necessary to differentiate clinical manifestation. Minicircle kDNA can exist at around 10,000 copies per LD cell and this may vary among *Leishmania* species or between isolates of a single species [29,42,43]. But importantly, the copy number of minicircle kDNA is relatively stable in amastigotes infecting a single patient [27,44]. Furthermore, no significant stage specific differences in copy number between amastigotes and promastigotes is evident, which justifies the use of standard curves generated from promastigote-derived DNA for the quantification of amastigotes of that species or strain [43]. In this study, the same standard genomic DNA of LD was used in both LD-qPCR and Q-RPA assays for comparative evaluation of quantitative features. Since all the test samples could not be assayed against a single dilution series due to limitation of slots in both PCR and RPA readers, Ct and Tr as well as template load per reaction was extrapolated from the respective standard series curves. This produced small differences in correlation coefficient values between threshold points (r = 0.89) and respective template loads (r = 0.87). DNA extracted from clinical specimens that involved two different sample matrices (blood for VL, skin biopsy for PKDL) yielded no differences in sensitivity. The absolute sensitivity of the Q-RPA method was determined against the real time LD-qPCR method, whereas absolute agreement of the method with C-RPA was observed for the outcome of the clinical test samples. Notably, we observed a significantly higher parasite load quantified by the Q-RPA assay than that by the LD-qPCR assay (Figure 4C). This is plausible since we considered the equivalent weighted measure of 1 parasite as 100 fg standard genomic DNA as per our established LD-qPCR assay that targets a repetitive region on the *Leishmania* chromosome [30], whereas there could also be difference in relative copy number of the extrachromosomal kDNA sequences between standard and clinical samples. Nevertheless, the correlation matrices between the two assays suggest that as an alternative to the LD-qPCR, the quantitative RPA assay has the potential to be used for monitoring of parasitemia levels in individual patients with different clinical manifestations. In the next phase of the study, evaluation with expanded number of samples from individuals with different clinical manifestations and transition (such as relapsed or cured) will be performed to validate the quantitative Q-RPA assay.

## 5. Conclusions

Quantitative metrics may be used to estimate infectious disease burden and/or offer a longitudinal assessment of response to treatment regimens used in disease mitigation efforts. Our target in this study was to evaluate the quantification capability of RPA to convey clinical significance in PONT field conditions and in particular resource-poor settings avoiding the use of any supplementary machinery such as continuous stirrer, vortex, and centrifuge. In this study, all the calculations in derivation of outcome/quantification from raw fluorescence data were performed using excel sheets and simple statistical formulas only (Figure S3). By defining the amplification trend and heuristically evaluating it with relative genomic and clinical samples, we showed that the Q-RPA assay has quantification potential for monitoring parasitemia. Further studies with a larger sample size and potentially with RPA assays for other pathogens of interest can be undertaken for fine tuning and validation of the approach described here. Moreover, the mathematical considerations to attain an outcome at each read-point potentiates the Q-RPA data analysis in a simple and automatically programmable interface for real time monitoring and end point quantification.

## Figures and Tables

**Table 1:** Reproducibility of the Q-RPA assay.

Inter-Assay Variation of Tr in Seconds (NFW)						Inter-Assay Variation of Tr in Seconds (CSB)						
Parasite load	Assay 1	Assay 2	Mean	SD	CV (%)	Parasite load	Assay 1	Assay 2	Assay 3	Mean	SD	CV (%)
1 × 10^4^	301.32	302.02	301.67	0.50	0.17	2.5 × 10^2^	262.67	255.33	273.10	263.70	8.93	3.39
1 × 10^3^	360.45	357.02	358.73	2.43	0.68	2.5 × 10^1^	304.77	297.56	311.60	304.64	7.02	2.30
1 × 10^2^	413.19	393.14	403.16	14.18	3.52	2.5 × 10^0^	341.96	354.85	350.90	349.24	6.60	1.89
1 × 10^1^	452.70	462.87	457.79	7.19	1.57	2.5 × 10^−1^	395.85	403.17	395.08	398.03	4.46	1.12
1 × 10^0^	496.73	500.12	498.42	2.40	0.48	2.5 × 10^−2^	459.90	455.29	457.57	457.58	2.30	0.50
1 × 10^−1^	555.79	556.92	556.36	0.80	0.14							
1 × 10^−2^	610.96	626.92	618.94	11.29	1.82							

## Supplementary Materials

The following are available online at https://www.mdpi.com/article/10.3390/diagnostics11111963/s1, Supplementary S1: Theoretical considerations, Supplementary S2: Curation of Q-RPA amplification data for detection and quantification, Figure S1: Schematic of onset of an RPA reaction step in a globule, Figure S2: Schematic representation of amplification trends in various RPA approaches, where reaction globules are either template-positive (yellow) or template-negative (green), Figure S3: Q-RPA variable generation from machine read data in MS-excel sheet, Table S1: Information of test samples used in E-phase threshold determination, Table S2: Test sample information and results obtained in LD-qPCR, Q-RPA and C-RPA assays.
