# Supplementary material for: Development of Quantitative Rapid Isothermal Amplification Assay for Leishmania donovani
Source: Diagnostics (Basel). 2021 Oct 22;11(11):1963. doi: 10.3390/diagnostics11111963 (PMC8625035; doi:10.3390/diagnostics11111963)
Supplement: Supplementary file 1 [file diagnostics-11-01963-s001.zip › Supplemental information.pdf]

Supplementary Information

Table S1. Information of test samples used in E-phase threshold determination. Samples were classified based on range of Ct values (color-shaded).

Sample ID	LD-qPCR Ct	E-phase duration in seconds (approximate)
**1-041**	30.825	615
**1-042**	30.915	605
**1-021**	31.07	545
**1-012**	31.28	550
**1-016**	31.57	550
**1-017**	31.98	555
**1-007**	32.28	595
**1-029**	33.4	595
**1-038**	33.76	565
**1-034**	33.85	545
**1-027**	33.97	565
**1-035**	34.15	595
**1-044**	34.16	565
**1-006**	34.31	565
**1-048**	34.35	595
**1-026**	34.43	575
**1-047**	34.48	545
**1-030**	34.55	605
**1-025**	34.61	550
**1-010**	34.9	535
**1-004**	35.05	525
**1-050**	35.05	565
**1-008**	35.4	535
**1-013**	35.65	530
**1-009**	35.73	545
**1-039**	36.83	535
**1-005**	36.94	450
**1-015**	37.05	565
**1-020**	37.38	470
**1-033**	38.92	525
**asy-01**	-	30
**asy-02**	-	50
**asy-03**	-	50
**asy-04**	-	80

**asy-05**	-	60
**asy-06**	-	30
**asy-07**	-	40
**asy-08**	-	130
**asy-09**	-	100
**asy-10**	-	70
**asy-11**	-	60
**asy-12**	-	40
**asy-13**	-	100
**asy-14**	-	210
**asy-15**	-	200
**asy-16**	-	80
**asy-17**	-	130
**asy-18**	-	60
**asy-19**	-	90
**asy-20**	-	80
**asy-21**	-	40
**asy-22**	-	130
**asy-23**	-	200
**asy-24**	-	90
**asy-25**	-	100
**asy-26**	-	60
**asy-27**	-	50
**asy-28**	-	40
**asy-29**	-	60
**asy-30**	-	80

Table S2. Test sample information and results obtained in LD-qPCR, Q-RPA and C-RPA assays. Samples were classified based on range of Ct values (color-shaded)

Sample ID	Patient type/DNA source	LD-qPCR (Ct)	C-RPA Tt (seconds)	Q-RPA Tr (seconds)	Template load per reaction (Q-RPA)	Template load per reaction (LD-qPCR)	Repeated in E-phase cut-off step
**pk-21**	PKDL/skin punch biopsy	25.77	318	268.1887	3574.2703	1036.96	no
**pk-31**	PKDL/skin punch biopsy	28.07	342	294.5439	1611.5587	250.02	no
**pk-30**	PKDL/skin punch biopsy	29.78	480	327.2975	395.5728	68.14	no
**pk-27**	PKDL/skin punch biopsy	29.82	318	346.2408	337.8079	29.77	no

**pk-45**	PKDL/skin punch biopsy	29.99	360	364.4911	194.5880	63.72	no
**1-041**	VL/peripheral blood	30.82	283.8	348.6775	57.0750	25.27	yes
**1-042**	VL/peripheral blood	30.91	324	366.7608	32.5988	24.39	yes
**1-021**	VL/peripheral blood	31.07	421.2	314.857	203.8787	37.49	yes
**1-012**	VL/peripheral blood	31.28	363.6	381.092	50.0899	17.03	yes
**1-031**	VL/peripheral blood	31.77	318	371.1655	38.8844	24.09	no
**1-017**	VL/peripheral blood	31.98	442.8	385.1767	104.1347	5.22	yes
**pk-28**	PKDL/skin punch biopsy	32.25	318	399.1968	68.1658	6.04	no
**pk-06**	PKDL/skin punch biopsy	32.25	438	435.2529	5.3101	16.38	no
**1-007**	VL/peripheral blood	32.28	384	369.762	77.4059	16.21	yes
**pk-19**	PKDL/skin punch biopsy	33.02	342	426.5876	8.7242	9.9	no
**1-029**	VL/peripheral blood	33.4	400.8	403.8656	10.3298	8.6	yes
**1-045**	VL/peripheral blood	33.45	702	402.7065	21.8346	3.95	no
**pk-22**	PKDL/skin punch biopsy	33.73	402	452.9884	3.1023	3.4	no
**pk-25**	PKDL/skin punch biopsy	33.83	318	381.6445	49.0379	1.45	no
**1-043**	VL/peripheral blood	34.35	378	426.6306	7.6025	2.2	no
**1-048**	VL/peripheral blood	34.35	429	386.2104	41.1489	2.02	yes
**pk-04**	PKDL/skin punch biopsy	34.74	378	413.2267	14.5758	3.36	no
**pk-41**	PKDL/skin punch biopsy	34.76	360	428.5616	6.5038	1.25	no
**1-036**	VL/peripheral blood	35.2	582	370.9053	39.1833	2.74	no
**1-013**	VL/peripheral blood	35.65	460.8	461.2454	1.7468	0.94	yes
**1-009**	VL/peripheral blood	35.73	426	408.594	12.9257	1.84	yes
**pk-48**	PKDL/skin punch biopsy	36.56	480	522.3393	1.6488	0.9	no

**1-014**	VL/peripheral blood	36.71	318	537.8795	0.1627	0.28	no
**1-005**	VL/peripheral blood	36.94	528.6	467.486	2.2847	0.8	yes
**1-015**	VL/peripheral blood	37.05	381	517.3065	0.3077	0.14	yes
**1-023**	VL/peripheral blood	37.22	318	464.6856	2.0189	0.77	no
**1-020**	VL/peripheral blood	37.38	682.8	485.1867	5.0682	0.11	yes
**pk-46**	PKDL/skin punch biopsy	37.74	618	523.5132	0.3660	0.46	no
**pk-17**	PKDL/skin punch biopsy	37.92	540	534.1365	1.1543	0.44	no
**pk-36**	PKDL/skin punch biopsy	38.51	480	540.3335	0.2198	0.07	no
**pk-37**	PKDL/skin punch biopsy	38.64	558	457.6744	2.6916	0.09	no
**1-022**	VL/peripheral blood	38.67	318	500.2418	0.5220	0.37	no
