# Supplementary material for: Development of Quantitative Rapid Isothermal Amplification Assay for Leishmania donovani
Source: Diagnostics (Basel). 2021 Oct 22;11(11):1963. doi: 10.3390/diagnostics11111963 (PMC8625035; doi:10.3390/diagnostics11111963)
Supplement: Supplementary file 1 [file diagnostics-11-01963-s001.zip › Supplementary Materials.pdf]

Supplementary S1: Theoretical considerations

The RPA process initiates in a phase separated system formulated by a viscous crowding agent - a high molecular polyethyleneglycol (PEG). This prompts the reactions to occur within colloidal globules, where RPA proteins (ssDNA binding protein- Gp32, recombinase protein- UvsX and UvsY, Sau polymerase enzyme), primers, probe and template DNA can be localized to allow nucleic acid amplification. Microscopic observation suggests that oligonucleotides of as small as 15 nucleotides in length become trapped in these globules even in the absence of amplification [1]. The reaction starts with the formation of a recombinase-primer complex, which seeks for a sequence homology in double stranded DNA to perform the strand invasion at the cognate site and then translocate the primer molecule by hydrolyzing ATP, forming primer-DNA complex. The reaction is known to be heavily dependent on the presence of PEG, which prevents the spontaneous recombinase-primer disassembly that occurs in the presence of the single stranded binding protein (Gp32). The elongation of recombinase filament assembly is suggested to be one of the rate limiting steps in RPA reaction and a major contributor of delay time, while this time delay is much less sensitive to the initial DNA concentration (2). Once the recombinase-primer complex far exceeds the number of available template DNA substrate, typical polymerase mediated amplification of isothermal assay begins known as the growth phase, where the limited supply of DNA determines the rate of amplification and follows a roughly exponential trend. However, the viscosity of PEG renders slow diffusion of reagents through the reaction mixture and thus the steady state equilibrium is not applicable in the primer translocation reaction, particularly when the DNA substrate level is low [2].

$$\text{Recombinase•Primer} + \text{DNA} \rightleftharpoons \text{Recombinase•Primer•DNA} \xrightarrow[\text{ATP} \;\; \text{ADP+Pi}]{} \text{Primer•DNA} + \text{Recombinase}$$

To minimize the diffusion mediated influence caused by the crowding agents that can lead to non-uniformity in the amplification cascade across the globules, the default TwistDx protocol includes a vigorous mixing step after around 4 minutes [3], which is deemed recommended in reactions with low number of target sequences. While the time and speed to mix should be optimized depending on a number of factors including the length of the target amplicon, our preliminary observation (not shown) similar to that by Moody and colleagues [2] indicates that the mixing step introduces variability in results, particularly with regards to the threshold time appointment relative to the initial template concentration, the gap in real time data collection due to removal of tubes, and the possibility of higher rate of false negatives. The variability in results may emerge from the disturbance of crucial points of the amplification cascade. One instance could be pertaining to the primer translocation kinetics: in a high template load scenario, the translocation reaction in a single globule proceeds in forward direction amid diffusion effects, and a lot of DNA is being produced as a result. This, upon stirring, may or may not further enhance the DNA production rate depending on the reaction kinetics in post-stirred globules. On the other hand and in particular when the diffusion effects are still impeding a low template

reaction in a globule to formulate enough translocation assemblies to reach similar reaction kinetics in a given time, the low number of amplicons upon stirring can be disseminated to post-stirred globules that might be high, low or void of templates before stirring, thus shifting a globular translocation reaction to either more early or late phase than that before stirring - causing variability in amplification trends within the given reaction time.

Now, if it is considered that a uniformly mixed reaction contains RPA proteins, polymerase, oligonucleotides and dNTPs in similar ratios and distribution across uniformly sized globules, the only factors that will differentiate the trend of DNA production of a low template reaction from that of a high template reaction would be the concentration of templates in the globules as well as the number of template occupied globules. Nevertheless, the reaction kinetics in diluted samples is thought to be increasingly influenced by the initial diffusion effects (e.g., rate of movement of molecules, thermal convection etc.) that can be associated with concentration-dependent time delay [2,4]. In other words, the magnitude of amplicon production can compensate for the influence of the diffusion effect. While the rate and quantity of recombinase-primer complex formation is steady across globules and less sensitive to template concentration, it can be considered that the diffusion rate through the viscous crowding agent of a single template DNA of the same length, be it genomic or amplicon, is indifferent to high or low template reaction. In the initial stage of amplification, although the number of recombinase-primer complex are in greater densities in a globular compartment, these can only bind to proportional number of available template DNA. Thus it can be hypothesized that the diffusion rate in the initial RPA amplification cycle is inversely proportional to the number of templates, which provides a window of linear template-time relationship however briefly. We assume that the polymerase generates amplicons in this reaction window. If diffusion rate was constant, the rate of DNA production would be the only determinant of the exponential behavior- a hallmark of template based amplification. However, amplicons reduce the diffusion time by an order of magnitude during the continuation of the growth phase, and PEG-induced diffusion impediment starts causing progressively increasing differences between high and low template globules in the rate of template discovery by surrounding recombinase-primer complexes, thus producing a time delay in reaction kinetics (Figure S1). Hence, as the template-time relationship is more likely to be synchronized at the early growth phase, it can provide an interval window to monitor the relationship.

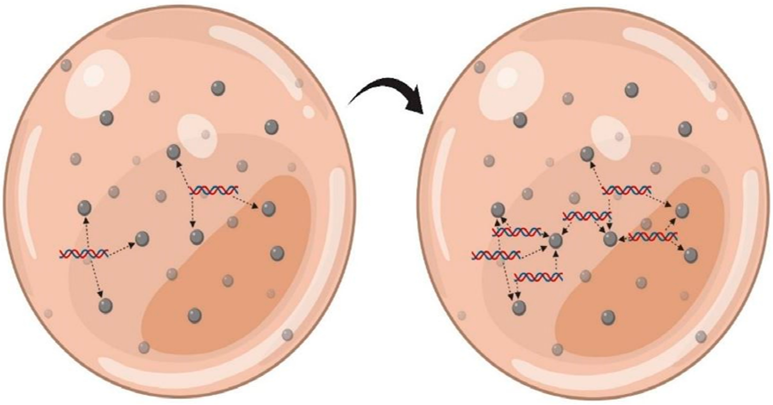

Figure S1. Schematic of onset of an RPA reaction step in a globule. Diffusion space and time for amplicons to bind recombinase-primer complex (indicated by grey balls) can be increasingly distinct with regards to the rate of amplicon production.

In earlier studies, mechanical instrumentation-aided continuous mixing has been proposed as a possible solution to diffusion effects, and some results support the methodological benefits in terms of faster time of amplification, increased sensitivity and repeatability [2,5]. Moody et al further suggested that a quantifying approach for the initial DNA concentration using RPA should require a mixing method with highly repeatable outcomes. However, this approach may need careful optimization of centrifugal speed as well as more instrumentation and power support. Another evolution of RPA approach is based on compartmentalized reaction principle where sample and reaction components are compartmentalized into many individual and parallelized reactions in small volumes such that each reaction contains one or no copy of the target DNA. The compartmentalization approaches that have been developed include digital plasma separation, centrifugal step emulsification, SlipChip technology and picoliter array based technology, as discussed elsewhere [6]. The basis of this reference-independent quantification approach lies in monitoring of the end-point amplification signal from compartmentalized single template reactions. In contrast to these approaches, we assumed that the inherently compartmentalized reaction principle of RPA can be harnessed to explain template-time relationship from its amplification trend. The possible amplification trend in the different RPA methodological variations described above can thus be varying (Figure S2).

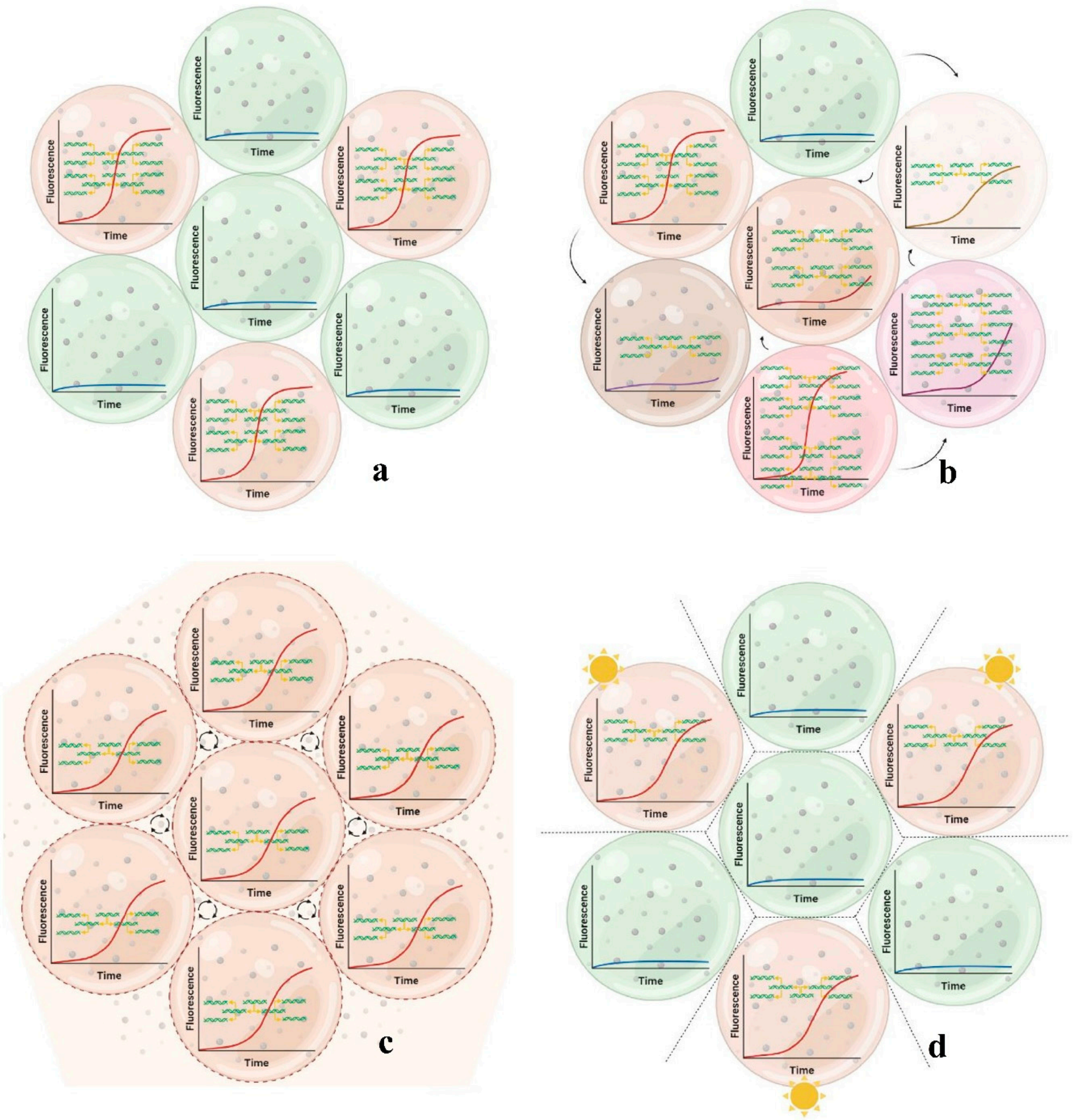

Figure S2. Schematic representation of amplification trends in various RPA approaches, where reaction globules are either template-positive (yellow) or template-negative (green). **a**. Unperturbed amplification in template-contained globules (yellow) follows particular reaction kinetics based on template number. **b**. Mixing at a break can potentially alter this kinetic by changing the template concentration in globules (indicated by different color). **c**. Continuous mixing can unify the reaction rate across all the temporary globules solving the diffusion effect. **d**. Mechanically compartmentalized reaction with single template provides end-point visual output.

Supplementary S2: Curation of Q-RPA amplification data for detection and quantification

The observable template amplification phase denotes positive change in the acquisition of total fluorescence number over time (F_t) (i.e., $F_{t+1} - F_t \geq 1$) for a consecutive period (Ap) of read-points. We hypothesized that the Ap separates the background/noise floor. Noise-unbiased sample fluorescence (F_n) at any t-th read-point thus can be derived as:

$$F_n = (F_t / (F_{background} / N))$$

Here, F_t is the cumulative fluorescence at any timepoint and $F_{background}$ is the central (i.e., median) value of background fluorescence of any test sample that approaches the Ap. Assay noise, N, denotes the mean fluorescence value (+2 SD) of assay negative control for total duration.

The fold change ratio in fluorescence acquisition rate (R_{fold}) for any given t-th read-point above the noise floor can thus be calculated as-

$$R_{fold} = ((F_n - N) / (F_{n-1} - N))$$

For the reaction to assert exponential behavior, Rfold must be greater than 1.0 fold for a consecutive period of read-points. This hypothetical exponential period where Rfold is consecutively increasing by more than 1.0 fold was thus denoted as E-phase. The threshold record-time (Tr) would thus correspond to a time point that belongs to E-phase.

To test whether the duration of the R_{fold} derived E-phase (the longest consecutive period where R_{fold} is > 1.0) can differentiate a positive detection from a negative outcome, 30 LD-qPCR confirmed LD positive and LD negative archived DNA samples were assessed. The positive samples were distributed on the basis of parasite loads determined by LD-qPCR assay and corresponded to low (<32), moderate (32 to 35) and high (>35) Ct values. The outcomes were fitted in a ROC curve to determine the optimal cut-off time period (in seconds) of the E-phase for differentiation between a true template amplification from template negative outcome.

To establish the minimum number of parasite genomes detected by the assay, the serial dilution series of DNA of reference strain (*L. donovani* MHOM/IN/80/DD8) was assayed in two independent runs. To test further the assay analytical sensitivity in mock clinical specimen, a spiked blood dilution series was assayed in three independent runs and variability among the replicates was estimated. From the noise optimized dataset which is not integer, Tr values of each sample in the dilution series were estimated on the basis of an arbitrary fluorescence level ($F_{n(arbitrary)}$) reached within the E-phase by all the dilutions in respective times. As discussed before, a suitable cut-off fluorescence level that likely associates the time needed for a given number of template to reach this fluorescence would ideally lie in the early growth phase of the exponential window. This ($F_{n(arbitrary)}$) value that could cover most log-linear template-time relationships was estimated statistically by fitting and comparing corresponding Tr (of several ($F_{n(arbitrary)}$) values) at which all the reactions of a dilution series reached to these particular fluorescence level, against the initial template load. It is to be noted that the differentiator was calculating from normalized fluorescence values that are essentially non-

integers under the consideration that all the hypothetical fluorescence reads within any reading interval are evenly dispersed.

$$Tr = ((T_{unit} / (F_{n(t+1)} - F_{n(t)})) * (F_{n(arbitrary)} - F_{n(t)})) + t_t; \text{ where } t \in \text{E-phase}$$

Here, T_{unit} indicates unit read-time (i.e., 10/20 seconds), $F_{n(arbitrary)}$ represents an arbitrarily set fluorescence value that lies proportionately between two noise-optimized normalized reads $F_{n(t+1)}$ and $F_{n(t)}$. t_t is the time point that corresponds to $F_{n(t)}$.

A schematic of an exemplary Q-RPA variable generation from machine read data is shown in Figure S3.

By using the normalized fluorescence values, the fluorescence reads starting above noise level and about the $F_{n(arbitrary)}$ differentiator was transformed as a function of Tr to fit to a deviated model of what has been previously described mathematically (4). In this generalization, exponential amplification is considered to initiate at the Tr under a hypothetically constant reaction condition.

$$r_a = r_{max} / (1 + (e^{kTr} * e^{-ky}))$$

Here, r_{max} represents the maximum fluorescence that can be achieved in an assay run (i.e., a value that is asymptotically approached at the terminal end of the reaction) and "y" is the total time that has lapsed at a read-point above noise level. "k" represents average RPA amplicon generation (fluorescence acquisition) rate in unit read-time and is expressed in $second^{-1}$ (i.e., ((ΣR_{fold}/ Σ number of R_{fold} read points) – 1)/unit read-time).

Standard curves were generated from the log-linear regression analysis after plotting Tr values against the parasite loads that represent corresponding dilution. Next, sample Tr was extrapolated from the standard curve, as the time needed to reach the differentiator value (i.e., $F_{n(arbitrary)}$) set for the calculation of Tr values of the dilution points.

For quantification, amplification factor (F_{amp}) of RPA was estimated from the standard curves as-

$$F_{amp} = 10^{(-1/\text{standard curve slope})}$$

Finally, absolute template number in sample with regards to standard was estimated by the formula:

$$\text{Absolute template number} = F_{amp}^{\,(\text{standard curve intercept value - sample Tr})}$$

Test Sample							Non-template control			
Read-point (second)	RFn	Amplification period (Ap)		Noise optimized RFn	R-fold	E-phase	Read-point (second)	RFn	Amplification period (Ap)	
20	374	0		209.4763169	0		20	200	0	
40	377	3		211.1566083	0.6		40	197	-3	
60	376	-1		210.5965111	1.222222		60	196	-1	
80	379	3		212.2768025	0.454545		80	196	0	
100	381	2		213.3969967	0.2		100	197	1	
120	381	0	Sample	213.3969967	1	No	120	198	1	
140	382	1	Background	213.9570938	-1		140	199	1	
160	383	1		214.5171909	3		160	199	0	
180	383	0		214.5171909	1		180	200	1	
200	383	0		214.5171909	1		200	200	0	
220	384	1		215.077288	1.666667		220	201	1	
240	384	0		215.077288	1		240	201	0	
260	386	2		216.1974822	1.8		260	201	0	
280	388	2		217.3176764	1.444444		280	202	1	
**300**	392	4		**219.5580648**	1.615385	**Tr**	300	202	0	
**320**	404	12		**226.2792301**	2.142857	**position**	320	202	0	
340	428	24		239.7215606	2.066667		340	203	1	
360	473	45		264.9259302	1.967742		360	202	-1	
380	544	71		304.6928246	1.775956		380	202	0	
400	637	93		356.7818553	1.572308		400	202	0	
420	743	106		416.1521484	1.414873		420	203	1	
440	859	116		481.1234124	1.320885		440	203	0	
460	981	122		549.4552591	1.255497		460	203	0	Assay
480	1105	124		618.9073	1.206839		480	203	0	Background
500	1232	127		690.0396323	1.175536		500	204	1	
520	1360	128		761.7320616	1.1505		520	204	0	
540	1486	126		832.3042967	1.128769		540	204	0	
560	1614	128		903.996726	1.11589	Yes	560	204	0	
580	1741	127	Ap	975.1290583	1.103043	(length =	580	205	1	
600	1868	127		1046.26139	1.093417	640	600	205	0	
620	1995	127		1117.393723	1.085436	seconds)	620	206	1	
640	2119	124		1186.845764	1.076852		640	207	1	
660	2239	120		1254.057416	1.069065		660	207	0	
680	2359	120		1321.269069	1.064603		680	208	1	
700	2476	117		1386.80043	1.059166		700	208	0	
720	2590	114		1450.6515	1.054428		720	209	1	
740	2703	113		1513.942472	1.051166		740	210	1	
760	2814	111		1576.113251	1.047814		760	210	0	
780	2922	108		1636.603738	1.044399		780	211	1	
800	3025	103		1694.29374	1.040543		800	211	0	
820	3126	101		1750.863547	1.038207		820	212	1	
840	3224	98		1805.753064	1.035708		840	212	0	
860	3317	93		1857.842094	1.032718		860	212	0	
880	3410	93		1909.931125	1.031681		880	213	1	
900	3497	87		1958.659573	1.028727		900	213	0	

Figure S3. Q-RPA variable generation from machine read data in MS-excel sheet. Threshold time (Tr) position corresponds to a noise optimized fluorescence (Fn) value that is set for derivation of standard curve.

References

1. Powell, M.L.; Bowler, F.R.; Martinez, A.J.; Greenwood, C.J.; Armes, N.; Piepenburg, O. New Fpg probe chemistry for direct detection of recombinase polymerase amplification on lateral flow strips. *Analytical Biochemistry* **2018**, *543*, 108-115, doi:https://doi.org/10.1016/j.ab.2017.12.003.
2. Moody, C.; Newell, H.; Viljoen, H. A mathematical model of recombinase polymerase amplification under continuously stirred conditions. *Biochemical Engineering Journal* **2016**, *112*, 193-201, doi:https://doi.org/10.1016/j.bej.2016.04.017.
3. TwistDx. TwistAmp® COMBINED INSTRUCTION MANUAL. 2014.
4. Gordon, M.I.; Klemer, D.P.; Fuller, S.L.; Chang, J.H.; Klemer, D.R.; Putnam, M.L. Mathematical modeling of a real-time isothermal amplification assay for *Erwinia amylovora*. *Engineering Reports* **2019**, *1*, e12047, doi:https://doi.org/10.1002/eng2.12047.
5. Kalsi, S.; Valiadi, M.; Tsaloglou, M.-N.; Parry-Jones, L.; Jacobs, A.; Watson, R.; Turner, C.; Amos, R.; Hadwen, B.; Buse, J., et al. Rapid and sensitive detection of antibiotic resistance on a programmable digital microfluidic platform. *Lab on a Chip* **2015**, *15*, 3065-3075, doi:10.1039/C5LC00462D.
6. Lobato, I.M.; O'Sullivan, C.K. Recombinase polymerase amplification: Basics, applications and recent advances. *TrAC Trends in Analytical Chemistry* **2018,** *98,* 19-35, doi:https://doi.org/10.1016/j.trac.2017.10.015.
